# Erectile dysfunction and sex hormone changes in chronic obstructive pulmonary disease patients

**DOI:** 10.1186/2049-6958-8-66

**Published:** 2013-10-09

**Authors:** Hasan Kahraman, Bilal Sen, Nurhan Koksal, Metin Kilinç, Sefa Resim

**Affiliations:** 1 Department of Chest Diseases, Faculty of Medicine, Kahramanmaras Sutcu ImamUniversity, Yörükselim mah Hastane cad No 32, Kahramanmaras 46050, Turkey; 2 Department of Chest Diseases, Ondokuzmayis University, Faculty of Medicine, Samsun, Turkey; 3 Department of Biochemistry, Faculty of Medicine, Kahramanmaras Sutcu ImamUniversity, Kahramanmaras 46050, Turkey; 4 Department of Urology, Faculty of Medicine, Kahramanmaras Sutcu Imam University, Kahramanmaras 46050, Turkey

**Keywords:** Aging, COPD, Erectile dysfunction, Sexuality, Testosterone

## Abstract

**Background:**

The prevalence of sexual dysfunction in patients with COPD is high and its significance has not been sufficiently stressed. The aim of this study is to investigate the incidence of erectile dysfunction (ED) and the factors affecting its frequency in COPD patients.

**Methods:**

Seventy patients with COPD and 68 healthy volunteers were included in the study. The International Index of Erectile Function questionnaire was used to evaluate ED, and the Beck Depression Inventory was used to evaluate depression.

**Results:**

The smoking rate was higher and oxygen saturation (SaO_2_) and body mass index (BMI) were lower in the COPD group. Blood tests revealed higher levels of follicle stimulating hormone (FSH), luteinizing hormone (LH), and estradiol. Testosterone level was lower but it was not statistically significant. Various degrees of ED were detected in 78.6% of COPD patients and 55.8% of the controls. Depression was more common in the COPD group. There was a negative correlation between forced expiratory volume in 1 sec (FEV1) level and ED and between SaO_2_ and ED in the COPD group. A positive correlation was noted between age and ED in both groups. No significant correlation was found among hormonal status and FEV_1_, ED, depression, SaO_2_, or BMI.

**Conclusions:**

The present study provides further confirmation that COPD is a risk factor for erectile dysfunction. When establishing a treatment plan for improving the pulmonary function of COPD patients, sexual dysfunction and depression, which are usually neglected but diminish quality of life, should also be addressed.

## Background

Chronic Obstructive Pulmonary Disease (COPD) is a chronic, treatable, and preventable disease characterized by persistent airflow limitation. It occurs as a result of long-term exposure to harmful particles or gases (mainly cigarette smoke) that lead to increased inflammatory response in the airway [1]. The inflammation seen in COPD is not limited to the lungs and has systemic aspects [2]. Comorbidities are defined as one or more concomitant diseases either directly or indirectly, associated with COPD [3] and they include cardiovascular diseases, malnutrition, skeletal muscle dysfunction and loss, cachexia, osteoporosis, anaemia, lung cancer, gastroesophageal reflux, diabetes, metabolic syndrome, obstructive sleep apnea, depression, and anxiety [1,3,4]. Systemic disorders lead to decreased libido and erectile dysfunction (ED) by affecting the sexual function in males. Hormonal imbalances in systemic disorders are thought to arise from the testes or hypothalamo–hipophyso–testicular pathway [2]. Androgens rise to peak levels at around the ages of 20–40 and start to decline after 40 years of age in males [5]. Symptoms of androgen deficiency can include depression, anger, muscle and joint pain, anxiety, sleep disorders, fatigue, poor concentration and memory, decreased libido, ED, and reduced ejaculate output volume and speed [6]. ED is defined as a permanent insufficiency in achieving and/or pursuing an adequate and necessary erection in order to have satisfactory sexual activity [7]. Loss of sexual desire and function have been associated with decreased testosterone levels [8-12], which, along with a decrease in libido and erectile function in males, are also caused by ageing [12,13]. Low levels of testosterone and ED have been reported in males with respiratory diseases, such as COPD, asthma, and obstructive sleep apnea syndrome [9,14]. Dyspnea, coughing, muscle weakness, and diminished physical activity are among the major causes of decreased sexual activity in patients with COPD [15].

During the evaluation of COPD patients, physicians generally ignore some significant comorbidities, such as sexual dysfunction. A previous study demonstrated that 87% of patients with COPD do not discuss their sexual problems with their physicians and 78% do not share these problems with their wives [16]. Few studies have investigated ED incidence in patients with COPD, and this topic remains a neglected area of research. The aim of this study is to assess the causes and frequency of ED in patients with COPD and to draw attention to this subject.

## Methods

This study was conducted on 70 COPD patients and 68 healthy volunteers who had been referred to the KahramanmarasSutcu ImamUniversity, Medical School Pulmonary Disease Clinic and the AfsinStateHospital, Pulmonary Clinic. The approval for this study was obtained from the Ethical committee of the University. All participants signed an informed consent form prior to the study. Healthy volunteers were patients who had been referred to the Pulmonary Disease Clinic because of cough and chest pain of less than 4 weeks. They had no history of any chronic disease, and no other serious health problem was detected after the physical, laboratory, and radiological examinations. Patients who had coronary heart disease, diabetes mellitus, history of alcoholism, impaired health, or who were receiving any hormonal or psychological treatments were excluded from the study. The selected patients filled out a questionnaire in order to identify their socio-demographic status. Body mass index (BMI) and oxygen saturation (SaO_2_), measured from the finger, were recorded.

### Pulmonary function tests

A pulmonary function test (PFT) was performed in compliance with American Thoracic Society criteria using a spirometry device (ZAN 500; nSpire Health GmbH, Oberthulba, Germany) [17]. Post-bronchodilator forced expiratory volume in 1 sec (FEV_1_), forced vital capacity (FVC), and FEV_1_/FVC were measured. A post-bronchodilator FEV_1_/FVC ratio less than 70% of the predicted value confirmed the diagnosis of COPD. The severity of COPD was graded based on the GOLD classification as mild, moderate, severe, and very severe [1].

### International index of erectile function

The International Index of Erectile Function (IIEF-5) is a self-applied five-question index developed by Rosen et al. [18] that is intended to evaluate erectile function. The maximum score is 25 points, and classification is as follows: 1–11 points, moderate to severe; 12–21 points, mild; and 22-–25 points, no ED. Severity was assessed as follows: 0, no ED; 1.degree, mild ED; 2.degree, mild to moderate ED; 3.degree, moderate ED; and 4.degree, severe ED [9,18].

### Biochemical analysis

Venous blood samples were obtained from all participants to measure total testosterone levels, follicle-stimulating hormone (FSH), luteinizing hormone (LH), and estradiol. The levels of testosterone, estradiol, and FSH were measured by enzyme-linked immunosorbent assay (ELISA) using a Thermo Scientific ® Multiskan FC (Finland), and LH was measured using the chemiluminescent method with an Immulite® 2000 (Siemens USA).

### Beck depression inventory scale

The Beck Depression Inventory Scale (BDI) is a 21-question, multiple-choice, self-applied inventory developed by Beck et al. [18]. It evaluates depression symptoms based on physical, emotional, and spiritual aspects. An assessment of the reliability and validity of the Turkish version was performed by Hisli et al. [19]. The cut-off score is 17 for this inventory, and a higher score indicates more severe depression.

### Statistical analysis

Findings were evaluated using the SPSS 17.0 software package. Data were expressed as the mean, range, and standard deviation. For the statistical assessment, independent *t*-test, Pearson correlation, one-way analysis of variance, and chi-square tests were applied, and p less than 0.05 was considered significant.

## Results

A total of 138 male subjects, 70 patients with COPD and 68 control volunteers, were included in the study. Socio-demographic information of all participants are presented in Table 1. The age ranges of the volunteers were 42–81 years for the COPD patients and between 45-80 years for the control group. The COPD group consisted of patients with a history of smoking. Out of these, 36 (51.4%) were former and 34 (48.6%) were current smokers. In the control group, 38 (55.8%) were non smokers, 20 (29.43%) had quit smoking, and 10 (14.7%) were current smokers. The duration of smoking abstinence was 4.7 ± 6.8 years in the COPD group. BMI was significantly lower in the COPD group compared to the controls (p = 0.003). There was a significant difference between the control group and the COPD one based on educational status (p = 0.017); however, no difference was found based on the living environment (p = 0.98) (Table 1).

**Table 1:** Socio-demographic findings of the male participants in both COPD and control group

	**COPD**	**Control**	**p value**
**Subjects (n)**	70	68	-
**Age (years)**	63.34 ± 10.13	59.77 ± 10.46	NS
**BMI (kg/m**^**2**^**)**	25 ± 4.43	27.8 ± 3.46	0.003
**Smoking (pack-years)**	37.67 ± 16.67	19.57 ± 14.32	0.000
**Education (%)**			
**Unschooled (%)**	35.7	19.1	0.017*
**Elementary school (%)**	45.8	47.1	
**High school or higher (%)**	18.5	33.8	

*BMI*, Body mass index; *NS*, not statistically significant.

*Unschooled group was statistically significant to High school or higher group.

Oxygen saturation was lower in the COPD group, and the difference was statistically significant (p = 0.000). Although total testosterone level was lower in the COPD group compared to the controls, the difference was not significant (p = 0.17). In the COPD group, FSH, LH, and estradiol levels were significantly higher compared to the control group (p = 0.024, p = 0.005, p = 0.000, respectively) (Table 2). The grading of the COPD patients was made according to the GOLD classification (Table 3).

**Table 2:** Pulmonary function and oxygen saturation findings of the male participants in both COPD and control group

	**COPD**	**Control**	**p value**
**SaO**_**2 **_**(mm Hg)**	93.24 ± 2.78	95.78 ± 1,92	0.000
**FVC (% pred)**	80.94 ± 18.9	94.77 ± 17.01	0.000
**FEV1 (% pred)**	60.64 ± 18.14	95.47 ± 17.53	0.000
**FEV**_**1**_**/FVC (%)**	57.66 ± 11.39	80.71 ± 6.18	0.000
**Testosterone (ng/mL)**	4.69 ± 2.59	5.35 ± 2.81	NS
**FSH mIU/mL**	12.54 ± 9.78	9.09 ± 6.53	0.024
**LH mIU/ml**	9.77 ± 6.16	6.64 ± 4.45	0.005
**Estradiol (pg/mL)**	39.1 ± 20.91	22.75 ± 14.38	0.000

*SaO*_*2*_, Oxygen saturation; *LH*, Luteinizing hormone; *FSH*, Follicle-stimulating hormone; *NS*, not statistically significant.

**Table 3:** Pulmonary function of the male COPD group according to GOLD classification

	**Predicted FEV**_**1 **_**(%) (mean ± SD)**	**n (%)**
**Mild**	86.36 ± 4.3	11 (15.7)
**Moderate**	64.52 ± 8.25	40 (57.1)
**Severe**	41.12 ± 6.1	16 (22.9)
**Very severe**	18.66 ± 5.5	3 (4.3)

**Figure 1:**
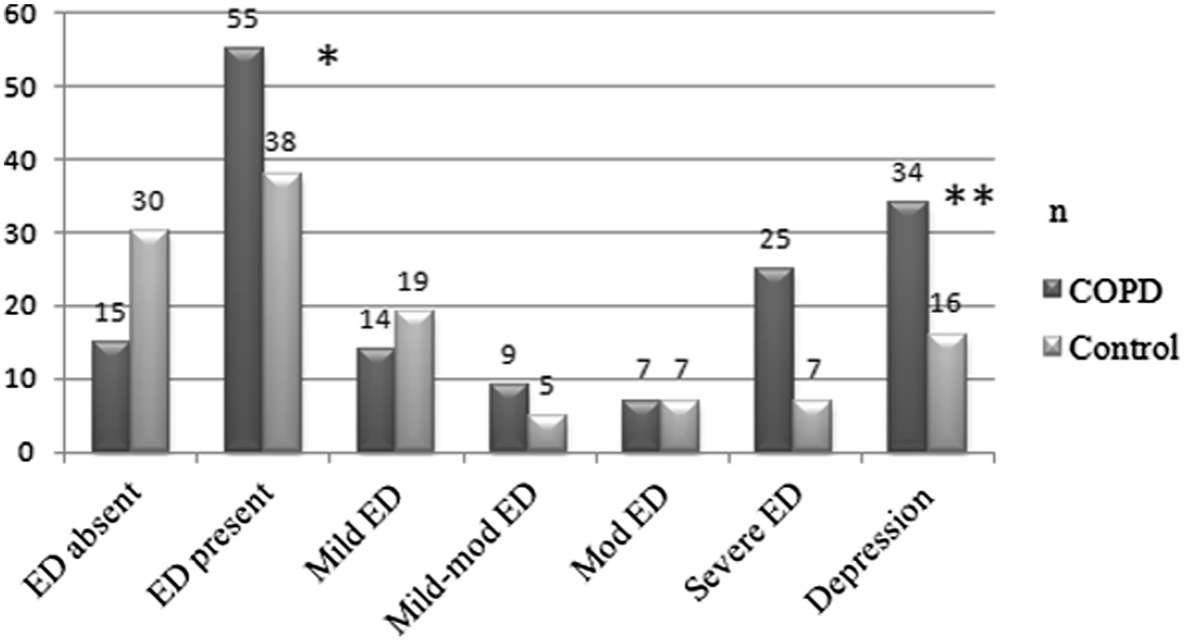
**Erectile dysfunction and depression features of male participants in the COPD and the control groups.** Mild to mod ED: mild to moderate ED; mod ED: moderate ED. BDI score was ≥17 in depression cases. * p = 0.000; ** p = 0,003.

**Figure 2:**
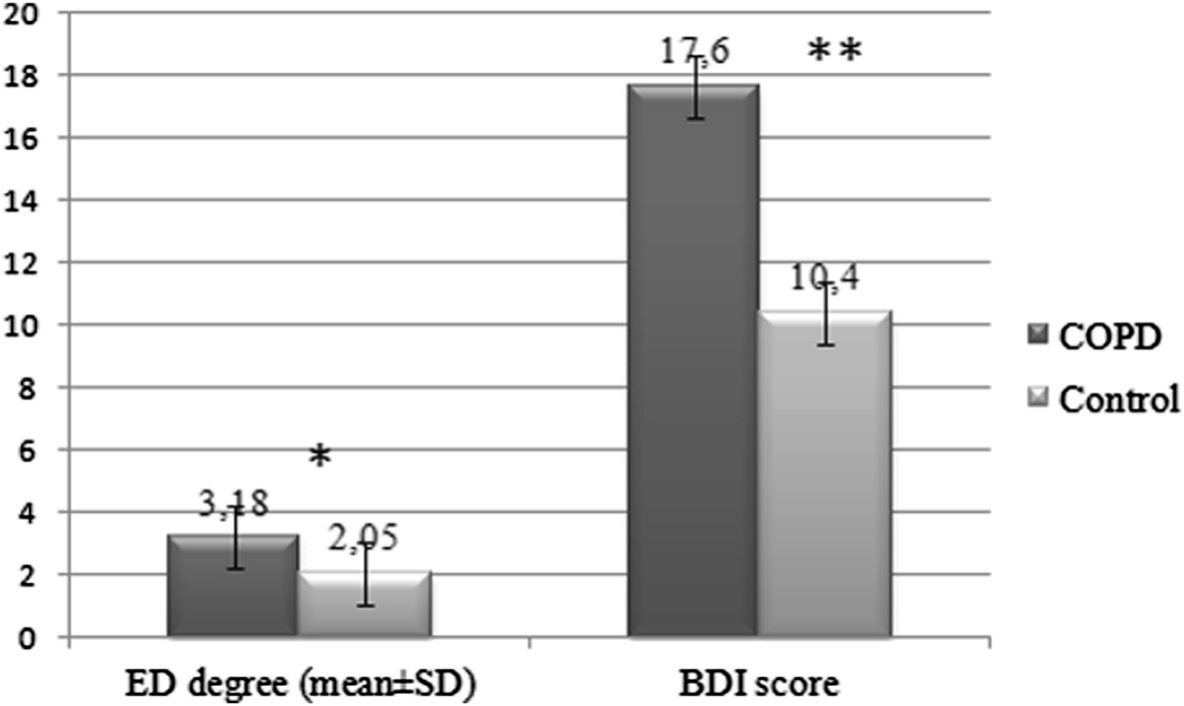
**Degree of erectile dysfunction and depression in the male participants of the COPD and the control groups.** BDI score, Beck Depression Inventory score. * p = 0.000.

Evaluation of IIEF-5 scores showed that 55 (78.6%) of the COPD patients and 38 (55.8%) of the controls had various degrees of ED, and the difference was statistically significant (p = 0.000) (Figure 1). The number of subjects who were considered to have depression (BDI score ≥17) was 34 (48.6%) in the COPD group and 16 (23.5%) in the control group. The difference was statistically significant (p = 0.003) (Figure 1). The mean degree of ED in the COPD group was higher than the controls (p = 0.000). The mean BDI score, demonstrating the depression status, was higher in the COPD group compared to the controls, and the difference was statistically significant (p = 0.000) (Figure 2).

### Correlations

In the COPD and control group, a moderate negative correlation was found between FEV_1_ and ED (r = −0.36, p = 0.00). There was no correlation between the level of FEV_1_ and BDI scores (r = −0.15, p = 0.39), but a negative correlation was found between FVC and BDI scores (r = −0.25, p = 0.03).In the control group, no correlation was demonstrated between FEV_1_ and ED, FEV_1_ and the BDI score, or ED and the BDI score.

In both COPD and control group, there was no significant correlation between hormone levels and FEV_1_, ED, depression, SaO_2_, and BMI (p > 0.005). However, a negative correlation was found between SaO_2_ and ED and between SaO_2_ and BDI in the COPD group (r = −0.26, p = 0.029; r = −0.37, p = 0.037, respectively). Likewise, a negative correlation was found between SaO_2_ and ED in the control group (r = −0.41, p = 0.001).

Age was correlated with both depression and ED in the control group, (r = 0.26, p = 0.044; r = 0.44, p = 0.00, respectively). On the other hand, there was a positive correlation between age and ED in the COPD group (r = 0.50, p = 0.00), while no correlation was detected between age and depression (r = −0.12, p = 0.29).

## Discussion

Sexuality is a lifelong necessity in order to pursue human well-being [9]. Factors including ageing and generally poor health can cause diminished sexual activity, libido, and ED. Physical and psychological problems, leading to morbidity, and medications can affect the sexual life of the patients [20]. A broad-based public study (Massachusetts Male Ageing Study) an ED prevalence as high as 52% in males between the fourth and seventh decade of life [21]. Dunn et al. demonstrated that 34% of males (mean age 50) within the general population complained about sexual problems and the most common complaint was ED [22]. Both studies showed that ED prevalence increases with age. In another study, ED prevalence was reported to be 7.6% between 40 and 49 years, 33.3% between 50 and 59 years, 70.2% between 60 and 69, and 90.1% over 70 years [23]. In our study we demonstrated that this ratio was 55.8% in the control group (mean age 59 years). In both groups (control and COPD) we documented a significant correlation between age and ED. The findings of all these studies support a strong association between age and ED.

COPD is not only a pulmonary obstructive disease but also a chronic illness, which presents with various comorbidities involving multiple organs [24]. Some of the well-defined comorbidities are muscle-joint diseases, hypertension, and cardiovascular, neurological, and gastrointestinal diseases [4,25,26]. As obvious from the GOLD guide [1] and the previously mentioned studies, sexual function disorders, such as ED, are not included in the comorbidities of COPD. Chronic diseases not only disrupt the natural course of daily life but also frequently cause deterioration of the sexual life [9]. In a study by Koseoglu et al. [27], ED was detected in 75.5% of 60 COPD patients (mean age 63 years), while other studies of patients with ED found 87% of 95 COPD patients [9], 86% of 50 COPD patients [28], and 72% of 90 patients [12]. In our study, the ED ratio was 78.6%, and the mean age was 63 years. Karadag et al. [9] did not found a significant difference between the COPD and the control groups based on the presence of ED, but in their study, the prevalence of moderate and severe ED was higher in the COPD group compared to the controls. Similarly, ED prevalence was significantly higher in our patient group. When we evaluated the severity of ED, the most common grade was severe ED (35.7%), which was an expected finding.

Sexual activity causes an increase in the cardiopulmonary load. Energy spent during orgasm is equal to the energy required for walking with a speed of 5–6 km/h or continuous stair climbing for 3–4 minutes [12,29]. Decreased exercise tolerance and fear of dyspnea may limit sexual activity [15]. Furthermore, in this patient population, misperceptions, ignorance, and poor physical or psychological status are common, and these factors contribute to sexual dysfunction [9]. Studies have reported that more severe disease has a higher ED frequency [9,27]. Likewise, the present study demonstrated a positive correlation between the severity of COPD and ED.

In males, testosterone is secreted mainly from the gonads. LH is released from the pituitary gland and controls the secretion of testosterone from Leydig cells. FSH contributes to an increase of testosterone secretion by inducing the maturation of Leydig cells [2]. In males testosterone gradually decreases with ageing and in parallel ED prevalence increases [30]. In a study investigating the association between plasma sex steroids and ED in elderly males, free testosterone was significantly lower and estradiol levels were higher, and this was associated with ED [31]. Another study demonstrated that testosterone levels of male COPD patients were lower than those of the controls [32]. Similarly, Karadag et al. [2] found lower levels of testosterone, an insignificant increase in FSH and LH, and a positive correlation between PaO_2_ and testosterone levels in COPD patients. Furthermore, they demonstrated a significant rise in LH and FSH and an important decline in testosterone levels during exacerbation in COPD patients. In our study, the levels of LH, FSH, and estradiol were significantly higher; however, the decline in testosterone levels was insignificant in the COPD patients. Similar to the study by Karadag et al. [2], our results showed no correlation between the testosterone level and ED score. In contrast to Karadag et al. [2], we did not demonstrate a significant correlation between via FEV_1_ score and hormone levels. This possibly was due to the lower number of severe COPD patients in our study.

Another factor leading to the sexual problems in COPD patients could be the low level of SaO_2_. ED incidence is reported to rise as the blood oxygen level decreases in healthy individuals ascending to a high altitude [33]. In a pioneer study conducted by Semple et al. [7], decreased libido was noted in nine patients and an absence of a morning erection was noted in seven out of 10 hypoxemic patients with COPD. Furthermore, with oxygen support therapy, serum testosterone levels increased with oxygen support therapy in all of these patients, sexual dysfunction improved in three patients, and the return of a morning erection was obtained in two patients. In our study, we found a strong significant correlation between hypoxemia and depression and also between hypoxemia and ED in the COPD patients. Our findings support the fact that hypoxia increases ED in COPD patients.

Anxiety and depression are among the most common comorbidities in COPD. Janssen et al. [34] demonstrated that depression incidence was 27% in 701 COPD patients and the incidence was 17.7% in the study of Turan et al. [35]. In our study, the depression incidence was 48.6% in the COPD group and 23.5% in the control group, and the difference was significant. We hypothesized that there would be a negative correlation between depression and FEV_1_; however, no significant correlation was found. On the other hand, we found a negative correlation between depression and FVC. We assume that an increased depressive status in the COPD patients contributed to the worsening ED.

Some limitations were noted during the evaluation of the results. The most important ones are the absence of the following parameters: the BODE index, the six-minute walking test, the partner status information, and the arterial blood gas test in both the study and the control group.

## Conclusions

GOLD guidelines do not contain information regarding sexual dysfunction in the comorbidities sections. Consequently, physicians who follow the GOLD guide usually do not ask patients whether they have any sexual complaints. This is an important limitation to patients’ quality of life. Sexual dysfunction and depression should be carefully questioned when recording the history of patients with COPD, and this information should be used in therapy planning.

## Availability of supporting data

The data set supporting the results of this article is included within the article.

## Abbreviations

BMI: Body mass index; COPD: Chronic obstructive pulmonary disease; ED: Erectile dysfunction; FSH: Follicle-stimulating hormone; LH: Luteinizing hormone; PFT: Pulmonary function test.

## Competing interests

The authors declare that they have no competing interests.

## Authors’ contributions

HK, BS participated in the performing the study. HK, NK conceived of the study, and participated in its design and coordination and helped to draft the manuscript. MK carried out biochemical analysis. SR performed the statistical analysis. All authors read and approved the final manuscript.
